# Disease-Specific Health Disparities: A Targeted Review Focusing on Race and Ethnicity

**DOI:** 10.3390/healthcare10040603

**Published:** 2022-03-23

**Authors:** Mark R. Cullen, Adina R. Lemeshow, Leo J. Russo, David M. Barnes, Yaa Ababio, Aida Habtezion

**Affiliations:** 1 Independent Researcher, 27766 Stirrup Way, Los Altos Hills, CA 94022, USA; 2 Global Medical Epidemiology, Worldwide Medical and Safety, Pfizer Inc., 235 E 42nd St., New York, NY 10017, USA; adina.lemeshow@pfizer.com (A.R.L.); leo.russo@pfizer.com (L.J.R.); david.barnes@pfizer.com (D.M.B.); yaa.ababio@pfizer.com (Y.A.); 3 Worldwide Medical and Safety, Pfizer Inc., 235 E 42nd St., New York, NY 10017, USA; aida.habtezion@pfizer.com

**Keywords:** race, ethnicity, health disparities, health equity, translational, United States, population, treatment

## Abstract

Background: Wide disparities in health status exist in the United States across race and ethnicity, broadly driven by social determinants of health—most notably race and ethnic group differences in income, education, and occupational status. However, disparities in disease frequency or severity remain underappreciated for many individual diseases whose distribution in the population varies. Such information is not readily accessible, nor emphasized in treatment guidelines or reviews used by practitioners. Specifically, a summary on disease-specific evidence of disparities from population-based studies is lacking. Our goal was to summarize the published evidence for specific disease disparities in the United States so that this knowledge becomes more widely available “at the bedside”. We hope this summary stimulates health equity research at the disease level so that these disparities can be addressed effectively. Methods: A targeted literature review of disorders in Pfizer’s current pipeline was conducted. The 38 diseases included metabolic disorders, cancers, inflammatory conditions, dermatologic disorders, rare diseases, and infectious targets of vaccines under development. Online searches in Ovid and Google were performed to identify sources focused on differences in disease rates and severity between non-Hispanic Whites and Black/African Americans, and between non-Hispanic Whites and Hispanics. As a model for how this might be accomplished for all disorders, disparities in disease rates and disease severity were scored to make the results of our review most readily accessible. After primary review of each condition by one author, another undertook an independent review. Differences between reviewers were resolved through discussion. Results: For Black/African Americans, 29 of the 38 disorders revealed a robust excess in incidence, prevalence, or severity. After sickle cell anemia, the largest excesses in frequency were identified for multiple myeloma and hidradenitis suppurativa. For Hispanics, there was evidence of disparity in 19 diseases. Most notable were metabolic disorders, including non-alcoholic steatohepatitis (NASH). Conclusions: This review summarized recent disease-specific evidence of disparities based on race and ethnicity across multiple diseases, to inform clinicians and health equity research. Our findings may be well known to researchers and specialists in their respective fields but may not be common knowledge to health care providers or public health and policy institutions. Our hope is that this effort spurs research into the causes of the many disease disparities that exist in the United States.

## 1. Introduction

Increasing attention has been paid over recent years to the gaping disparities in health status seen in the United States among people of lower socioeconomic status, and members of groups subject to discrimination based on gender, race, or ethnicity [1,2,3]. These demographics, as well as many others, are associated with a larger group of social determinants of health, whose effects have been dramatically illustrated during the COVID-19 pandemic, and of which workers in the health sector became acutely aware [4,5]. Moreover, pre-existing differences in longevity by race and neighborhood became widely discussed [1,6]. However, disparities in disease incidence and prevalence or differences isn severity and outcomes remain less well appreciated at the level of individual diseases. At the “bedside,” where daily decisions are made about preventive and therapeutic interventions for individual patients with specific health conditions, information about such differences between groups in the natural history of disease, prognosis, responses to available treatments, and potential for adverse outcomes is not readily accessible, and too rarely informs treatment guidelines or reviews used most by practitioners. At the research and development level, such disease-specific information rarely informs clinical trials and other translational research, leading to overly homogenous trial populations and lost opportunities to confront health disparities.

Some data do exist, and some have been published. There are myriad reports reviewing and assessing such data for common disorders. Most have derived population-level estimates using aggregate health care claims data, registries, or surveys of U.S. population samples such as the National Health Interview Survey (NHIS) or the National Health and Nutrition Examination Survey (NHANES) [7,8]. For less prevalent conditions, reviews of case series or local registries provide information on disparities, although of variable generalizability to the larger U.S. population. From these published sources, it is possible to establish that differences between U.S. non-Hispanic Whites (NHWs) and Black/African Americans exist in rates of occurrence and/or severity/prognosis for many disorders. Increasingly, evidence of disparities for populations of Hispanic and Asian origin are being reported as well, with appreciation for the substantial ethnic diversity within these groups, resulting in uncertainty of estimates for less prevalent conditions [9,10].

There is presently a lack of an easily accessible reference for routine “bedside” use by practitioners, service planners, policy makers and the like who may be aware of the social factors confronting their patients/clientele but lack evidence specific to individual conditions being managed. Most disease guidelines or online reference manuals address demographic aspects of the disease cursorily if at all, effectively rendering the information inaccessible. In other words, there is presently no summary of evidence highlighting what is known about specific disease disparities and targeting the most relevant literature at the condition level. The goal of this paper was to demonstrate, using a convenient small sample of diseases, an approach tackling this challenge; in an ideal world, such information would appear in every textbook, treatment guideline, and online tools used by practitioners.

In this targeted literature review, we present one approach for addressing a gap in data on health disparities confronting Black/African Americans and Hispanics that could be addressed within the health care system, and we focus on the set of diseases in Pfizer’s preventive and therapeutic pipeline. Our primary goal in this paper is to capture and summarize the published evidence of racial and ethnic disparities in the United States for this set of conditions so that this knowledge becomes readily available “at the bedside.” Our hope is that this summary will stimulate new waves of health equity research at the disease level so that potential causes of disparities can be further identified, and these health disparities can be effectively addressed.

## 2. Materials and Methods

Disorders of interest were selected based upon Pfizer’s current therapeutic and vaccine development pipeline (https://www.pfizer.com/science/drug-product-pipeline; accessed on 1 March 2021), recognizing that there is nothing otherwise unique about these disorders for our illustrative purpose. Included were a group of common metabolic disorders and cancers, along with a spectrum of inflammatory conditions, dermatologic disorders, and some rare diseases. The infectious targets of vaccines under development were also explored. The 38 studied diseases are summarized in Table 1 below. We approached the process of covering a large swathe of clinical disorders as follows.

First, we conducted online searches in both Ovid and a general search in Google, the latter to capture public documents and reports that may not yet have appeared in the peer-reviewed literature. In Ovid, the following string of key words were used in conjunction with disease name: “disparities” AND “race OR ethnicity.” We restricted our search to sources in English, about humans, and published from 2010 through present (December 2021) to focus on the latest available data wherever possible. When necessary, we included reports that drew on data sources developed earlier. For a few diseases we extended the search to include older sources of high relevance identified in the selected papers; these pre-2010 publications are listed in the footnote of Table 1. From these results, we identified reports that focused on differences in the distribution of disease rates and outcomes between NHWs and Black/African Americans, and between NHWs and Hispanics. Four types of papers were considered of greatest relevance to the development of our “disparity matrix”:

(1) Reports of large and comprehensive U.S. surveillance programs, of which SEER (The Surveillance, Epidemiology, and End Results) for cancer is the best example.
(2) Studies using large and representative health care claims and electronic medical records data sets, often reporting on large fractions of the U.S. population, such as Medicare.
(3) National surveys with standardized sampling strategies for extrapolation to the U.S. population, such as NHANES and NHIS.
(4) Review articles that relied on one or more of these source-types.

Citations of the articles and reports serving as the primary sources of data are listed in Table 1 for each condition. Reviews that relied directly on other primary data sources, regional registries or surveys, and cohorts or case-series from a single institution or group of institutions, were available and were especially important for assessing differences in outcomes and severity for non-lethal conditions. Sources that failed to meet stricter criteria for being population-based are listed in the footnote of Table 1. In some instances, they were the only source of data (especially for rare diseases) or the only source for assessing differences in severity, regardless of disease prevalence.

To make the results of our review most readily accessible, we grouped our observations into a small number of categories. For incidence rates and point prevalence, we designated diseases as rare (incidence < 1 per 100,000 per year or prevalence <0.1%) with a score of “0”, uncommon (incidence 1 to <20 per 100,000 per year or prevalence between 0.1 and <1.0%) with a score of “1”, common (incidence 20–50 per 100,000 per year or prevalence 1–10%) with a score “2”, and very common (incidence >50 per 100,000 per year or prevalence >10%) with a score of “3”. For differences in disease rates between Black/African Americans and NHWs and between Hispanics and NHWs, we used a similar scale, using “0” for when the minority group had lower disease rates than NHWs, “1” for conditions in which the literature suggests comparable risk for Black/African Americans or Hispanics compared with NHWs and/or non-Hispanics, “2” when modest excess risk (i.e., not greater than two-fold) for the minority group appears established, and “3” when a significant excess risk (i.e., relative risk exceeds two-fold for rare diseases or greater than 20% excess for common ones) for the minority groups appears established.

We scored disparity in severity in the same general way, recognizing that this concept is intrinsically more complex than frequency, and incorporates differences in rates of complications, hospitalization, response to therapy, and survival. For these comparisons, “0” applies to those entities for which NHWs have been reported to do worse in some key measure than the minority group; “1” when reported severity was comparable between the minority group and NHWs; “2” when some suggestion emerges from the literature that the minority group has greater severity, and “3” when these differences are demonstrated quantitatively from a population-based source such as survival differences in SEER. If no data from the medical literature were identified for a given disease disparity, “ND” was entered into Table 1.

After primary review of each condition by one author, one other author undertook an independent review. Where differences in the interpretation of the literature emerged, agreement on a final score was reached after discussion.

## 3. Results

A total of 38 conditions were reviewed for potential disparities comparing Black/African Americans vs. NHWs and Hispanics vs. NHWs. The list included 8 cancers, 11 inflammatory diseases, 6 common medical disorders, 8 rare and/or congenital diseases, and 5 vaccine-targeted conditions from the Pfizer pipeline. The list is presented along with the results in Table 1.

For Black/African Americans, 29 of the 38 disorders revealed an excess in incidence, prevalence, or severity, or suggestion of such a disparity. Aside from sickle cell anemia, Black–White differences were identified for multiple myeloma [11,12], hidradenitis suppurative [13], and lupus [14,15,16,17,18,19,20] among others. None of the conditions assessed had evidence of less severity for Black/African Americans [13]. As shown in Table 1, there was evidence of disparities for Hispanics in 19 entities. Notable differences from NHWs include NASH [21], obesity [22,23,24,25], and diabetes [26,27,28,29,30].

For Hispanics, there are larger gaps in knowledge of disease disparities than with Blacks. We found no substantive data on the incidence, prevalence, or disease severity comparing Hispanics to NHWs for 17 conditions, suggesting the possibility of further disparities being uncovered with additional research.

**Table 1:** Disease Areas of Pfizer’s Portfolio: Heat Map of Racial and Ethnic Disparities in Incidence, Prevalence and Disease Severity *.

Disease	Incidence/Prevalence	Black/AA Disparity Incidence/Prevalence	Black/AA Disparity in Severity	Hispanic Disparity Incidence/Prevalence	Hispanic Disparity in Severity	Citations
**Oncology**						
Prostate Cancer	3	2	2	0	1	[31,32,33,34,35]
Lung Cancer	3	2	2	0	0	[35,36,37]
Breast Cancer	3	0	2	0	1	[35,38,39,40,41,42,43]
Bladder Cancer	2	0	2	1	2	[44,45]
Colorectal Cancer	2	2	2	0	1	[35,46,47,48]
Head and Neck Cancer	2	2	2	ND	2	[49,50,51,52,53]
Melanoma	2	0	3	0	1	[54,55]
Multiple Myeloma	1	3	1	2	1	[11,12,56]
**Inflammation and Immunology**						
Ankylosing Spondylitis	1	0	3	1	2	[57,58,59,60]
Rheumatoid Arthritis	1	1	1	0	2	[61,62,63,64,65]
Osteoarthritis	3	1	2	1	2	[66,67,68,69,70,71,72,73,74]
Inflammatory Bowel Disease	1	0	1	0	0	[75,76,77,78,79,80,81,82,83]
Atopic Dermatitis	3	2	2	2	2	[84,85,86,87,88,89]
Psoriasis	2	0	2	0	ND	[90,91,92]
Hidradenitis Suppurativa	1	3	2	ND	2	[13,93,94,95,96]
Vitiligo	1	1	2	2	1	[97,98,99,100]
Alopecia Areata	2	3	2	1	2	[101,102]
Stasis dermatitis	2	0	2	0	ND	[103,104]
Systemic Lupus Erythematosus	1	3	2	2	2	[14,15,16,17,18,19,20]
**Internal Medicine**						
Diabetes Mellitus	2	2	3	2	2	[26,27,28,29,30]
Hypertriglyceridemia	3	0	1	2	ND	[105,106,107,108,109,110]
Obesity	3	2	ND	3	ND	[23,24,25]
Pain	3	0	3	0	2	[111,112,113,114]
Nonalcoholic Steatohepatitis	2	0	1	2	1	[21,115,116,117,118,119]
Pulmonary Arterial Hypertension	0	1	1	1	1	[120,121,122]
**Rare Disease**						
LMNA-Related Dilated Cardiomyopathy	0	3	ND	ND	ND	[123]
Growth Hormone Deficiency	0	1	2	1	ND	[124,125]
Hemophilia	0	0	ND	1	2	[126,127]
Duchenne Muscular Dystrophy	0	0	ND	ND	1	[128,129,130]
Focal Segmental Glomerulosclerosis	1	3	1	ND	ND	[131,132,133]
Achondroplasia	0	ND	ND	0	ND	[134,135]
Idiopathic Thrombocytopenic Purpura	0	0	2	ND	ND	[136,137,138]
Sickle Cell Anemia	0	3	ND	0	ND	[139,140]
**Vaccines**						
Lyme Disease	1	0	ND	0	ND	[141,142,143]
Clostridium Difficile Colitis	2	0	2	1	0	[144,145]
Streptococcus Pneumoniae	2	2	1	2	ND	[146,147,148,149,150,151]
Group B Streptococcus	2	2	2	2	ND	[152,153,154]
Meningococcal Meningitis	0	2	2	ND	ND	[149,155,156]

**Abbreviations:** AA = African American; ND = no data; NHW = non-Hispanic White; PY = person-year. **Scoring Legend:** Incidence (cancers and vaccines): 0 = <1 per 100,000 PYs; 1 = 1 to <20; 2 = 20 to 50 per 100,000 PYs; 3 = >50 per 100,000 PYs. Prevalence (all other diseases): 0 = <0.1%; 1 = 0.1 to <1%; 2 = 1 to 10%; 3 = >10%. Incidence/prevalence disparity: 0 = Lower than NHW; 1 = Equal to NHW; 2 = Modest excess compared to NHW; 3 = Significant excess compared to NHW. Survival disparity: 0 = Greater than NHW; 1 = Equal to NHW; 2 = Modestly worse than NHW; 3 = Significantly worse than NHW. Severity disparity: 0 = Lower than NHW; 1 = Equal to NHW; 2 = Suggestion in the literature more severely impacted than NHW; 3 = Quantitative evidence of greater severity than NHW.* For cancers, severity was assessed based on survival. For non-cancers, severity based on rates of complications, hospitalization, response to therapy, etc. (Source did not meet criteria for generalizability: [13,14,15,16,17,18,19,20,22,27,29,30,38,43,48,49,50,51,56,57,59,63,65,66,67,68,69,70,71,72,73,75,77,81,83,84,85,86,93,94,95,96,97,98,99,100,101,102,104,105,106,107,108,110,111,112,113,115,119,120,121,122,123,124,125,129,131,132,133,135,137,140,142,146,147,148,149,150,151,152,154,156]). (Source published prior to 2010: [104,131,135,142,149,154]).

## 4. Discussion

While the broad pattern of disease disparities by race and ethnicity in the United States has received increasing attention across the medical literature in recent years, and especially since the COVID-19 pandemic [157,158,159,160,161], few papers highlighting disparities for individual disease occurrence or severity have appeared in prominent journals aimed at health care providers, the institutions supporting them, and other parties in the health sector. The explicit purpose of this paper was to delineate disparities across a sample of diseases that are the targets for drug development and day-to-day medical care. We used a visual map to evidence differences in both frequency of occurrence and severity of diseases, comparing Black/African Americans with NHW and Hispanics with NHW populations, noting that we have not explored other important populations such as Native Americans, Asians, or Pacific-Islanders, each known to have unique risks as well. While many of the observations summarized in Table 1 are well known to researchers in their respective fields and specialists who focus on the diagnosis and treatment of the diseases with disparities, the darker cells on the heat map may not be common knowledge to health care providers and academic medical centers, public health institutions, and policy makers. The latter group, including those with responsibility for resource and facilities planning, as well as communication with the public is the core target audience.

Despite achieving our goal of visually displaying health disparities affecting Black/African American and Hispanic populations, we must recognize several limitations. First, we did not conduct a systematic review or a meta-analysis of any single disease. For the latter, the summary of broad evidence includes all available datasets meeting pre-specified criteria. While this approach is ideal for the establishment of the best available evidence for the effect of an exposure or intervention on an outcome, our aim here was solely to identify the best descriptive information available and did not lend itself to a meta-analytic approach. Contrasts between this paper’s findings and reports in the literature are most likely attributable to non-representative sampling or misclassification of the race or ethnicity being assessed as opposed to analytic methods or choices more indicative of meta-analytic reviews. Likewise, systematic reviews have become a standard approach to comprehensive literature reviews, with pre-specified criteria applied to an exhaustive exploration of available reports, taking a critical approach to each source. As our purpose here was to identify evidence of population-level disparities for easy accessibility by providers and service planners, systematic review was not warranted. As only large population-level sources satisfy the need for generalizable inference about disparities, most other available studies are of limited value for this purpose. Granular clinical details, which are of enormous value for follow-up investigations into the root causes of the identified disparities, are less valuable for the purposes of the current paper than the size and representativeness of the populations described. To answer the questions at hand, we needed to stand back from the proverbial elephant before attempting to analyze it. From a more practical perspective, very few rigorous population-based studies have been conducted for many diseases, especially those that are less common. Many disorders reported here are, in fact, “orphans” of this sort, but we have attempted to make sense of what is available, reserving the category “ND” for those rare instances when there is literally nothing to draw inference from.

While reports on disease rate differences based on population-level sampling were available for most conditions, including rare ones, studies of disease severity differences between groups proved more challenging. Large representative registries such as SEER provide quantifiable severity data by race and ethnicity (e.g., stage, survival), but most other sources are cross-sectional and lack standardized criteria for severity comparisons between groups. To assess such differences in less lethal conditions, such as lupus or hidradenitis suppurative, it was necessary to avail reports that did not meet the more stringent population-based criteria, but which represented reports from large clinical registries, regional or local surveys, cohort and panel studies, and, as a last resort, large case series. For obvious reasons, these sources sometimes differed in their findings. Whenever such conflicts arose, we erred on the side of increased sensitivity to detect a disparity, which future research may demonstrate was an over-reach, to limit the likelihood of missing disparities likely to emerge from future investigations. In each case we relied on such sources, we marked the references in Table 1 with an obelisk symbol to distinguish it from studies using population-based sampling.

Data on Hispanics were far more limited than for Black/African Americans, perhaps in part because “Hispanic” ethnicity, whether deemed of White or Black/African American race, comprises a particularly complex population with recent ancestries from multiple and diverse regions of the Americas, which complicates interpretability of results. The same problem makes inference difficult for Asian Americans and the group presently lumped together as Native Americans, Hawaiians, and Pacific Islanders, for whom data were deemed too sparse even to attempt review. It is important to note there is also genetic and cultural heterogeneity within the Black/African American category used in most health equity research. Compared with U.S. born individuals of African ancestry, foreign-born persons of African descent have been reported to have a different psychosocial context and different sociocultural determinants that influence their health status and disease risk [162]. For example, previous research has shown that Black Caribbean immigrants to the U.S. differ from African Americans on multiple measures of physical health status [163].

A final limitation was the scope of our work. We limited our review to a convenient sample of 38 disorders from the development pipeline of Pfizer. While these conditions range across a wide swathe of diseases and disease categories, our findings only reflect observations on those considered.

Notwithstanding these limitations, our approach combines a sharp focus on national population-based estimates, relying as little as possible on sources derived from local or regional observations, and a method that is reproducible for other diseases. We illustrate a pathway by which similar and extended information could be gleaned for every medical condition under care in the U.S.’s highly diverse population.

Recognition is only the first step in the path to amelioration of disparities. For few of the conditions assessed here has sufficient evidence emerged to identify root causes that can be remedied. For example, the study on the biology of hypertension in NHWs and African Americans has led to certain race-directed algorithms for pharmacologic interventions based on the likely population-level differences in genetic mechanisms [164,165]. With the advent of pharmacogenetics, other drugs that target population differences for some disorders will likely lead to an expansion of such testing, based on population-level risk of carrying a variant genotype, as has evolved for anticoagulants [166,167]. However, the evidence suggests genetic differences are likely to explain only a small portion of the observed disparities for many of these diseases. Although many would view the search for genetic differences extremely valuable for identifying new targets for therapy and more personalized treatment approaches, others may view it as a failure in assuming responsibility for the lower-hanging fruit: differential access to care and differing quality of care, and uneven distribution of resources; differences in patient knowledge and trust; and differential behaviors and social and physical environments. These factors have been identified as social determinants of health, which the WHO defines as “the conditions in which people are born, grow, live, work, and age” [168]. These social determinants, rather than group-level genetic or biologic differences, appear to be the main drivers of the extreme race and ethnic group disparities observed in COVID-19 outcomes [157,158,159,160,161]. That said, it is likely that at the individual disease level, the contributions of behavior, and social and physical environment will vary greatly. We have undertaken this review for the purpose of highlighting how to identify disease targets for more in-depth disparities research.

## 5. Conclusions

We hope that our paper will generate, first, greater knowledge and appreciation among frontline health care workers and their institutions of disease-specific race and ethnic group disparities, and second, more research documenting these disparities and identifying their root causes. From this perspective, Table 1 should be viewed as a “map” highlighting fruitful areas of focus for translational scientists, clinical investigators, and those who provide and pay for available and future interventions for these disorders. It is our hope that others will use the approach we have taken to continue to address gaps in our knowledge of the disparities landscape, while bolder colleagues begin the critical deep dives into the ripest areas for interventions to lessen these disparities.

Yet even with good science, solutions to address these disparities, short of radical redistribution of society’s resources, will likely depend first and foremost on recognition and acceptance of the inequity issue by the entire health industry—health care providers, insurers, payors, health care organizations, device and drug manufacturers, drug retail stores, and public policy makers all have potential roles. Pfizer, which undertook and supported this review, fully embraces this broad public responsibility, and invites other entities to examine disparities across its portfolios to identify knowledge gaps and motivate unified societal action.
